# Distribution of *Biomphalaria* sp. and other limnic mollusks in the state of Ceará: a potential effect of the São Francisco River Transposition Project

**DOI:** 10.1590/0037-8682-0316-2024

**Published:** 2026-02-09

**Authors:** José Damião da Silva, Alberto Novaes Ramos, Marta Cristhiany Cunha Pinheiro, Bárbara Morgana da Silva, Anderson Fuentes Ferreira, Maria Aparecida Alexandre de Sousa, Vivian da Silva Gomes, Gabriela Soledad Márdero García, Fernando Schemelzer de Moraes Bezerra

**Affiliations:** 1 Universidade Federal do Ceará, Programa de Pós-Graduação em Saúde Pública, Fortaleza, CE, Brasil.; 2 Universidade Federal do Ceará, Departamento de Saúde Comunitária, Fortaleza, CE, Brasil.; 3 Universidade Federal do Ceará, Departamento de Análises Clínicas e Toxicológicas, Laboratório de Pesquisa em Parasitologia e Biologia de Moluscos, Fortaleza, CE, Brasil.; 4 Secretaria da Saúde do Estado do Ceará, Coordenação do Programa de Vigilância e Controle da Esquistossomose, Fortaleza, CE, Brasil.; 5 Universidade Federal do Ceará, Programa de Pós-Graduação em Patologia, Fortaleza, CE, Brasil.; 6 Universidade Federal do Ceará, Programa de Pós-Graduação em Ciências Médicas, Fortaleza, CE, Brasil,

**Keywords:** Mollusca, Biomphalaria, Schistosomiasis mansoni, Epidemiology, Water resources

## Abstract

**Background:**

The São Francisco River Integration Project (PISF) may facilitate the spread of intermediate hosts of schistosomiasis to previously unaffected areas, potentially increasing disease transmission owing to human migration and environmental changes. This study aimed to describe the distribution of freshwater mollusk fauna along the river basins involved in the PISF in the state of Ceará.

**Methods:**

A descriptive cross-sectional study was conducted in three municipalities in the state of Ceará (Brejo Santo, Jati, and Mauriti) located along the Meta 2N axis of the PISF. Mollusks were collected from 21 water bodies near the resettlement areas (productive rural villages). Morphological identification of freshwater snails and screening for *Schistosoma mansoni* (*S. mansoni*) cercariae were also performed. Environmental variables were recorded, and the potential for transmission was assessed according to the Brazilian Ministry of Health guidelines.

**Results:**

Overall, 765 *Biomphalaria* specimens were collected, with *Biomphalaria straminea* being the only species identified. This genus occurred in all three municipalities and 85.7% of the surveyed water bodies. The other identified mollusk genera included *Melanoides*, *Drepanotrema*, *Physa*, *Pomacea*, and members of the class Bivalvia. No *S. mansoni* larvae were found, and only nonpathogenic trematode larvae were observed. Eight water bodies were classified as having a high epidemiological risk.

**Conclusions:**

The surveyed communities represent areas with the potential to sustain schistosomiasis transmission. These findings highlight the importance of developing intersectoral strategies to address the issue from a One Health perspective and to implement effective interventions in the areas impacted by the project.

## INTRODUCTION

The implementation of large-scale water projects invariably leads to significant environmental, economic, and sociocultural transformations that directly impact local dynamics. This process disrupts the daily lives of the affected populations, often resulting in displacement and changes in their livelihoods, particularly in terms of health and quality of life^1^ ^,^ ^2^.

In such projects, the creation of water reservoirs induces abrupt ecological shifts owing to the activities related to irrigated agriculture, livestock farming, fishing, and processing industries. These changes may foster the emergence and spread of diseases not previously endemic to the affected regions^2^ ^,^ ^3^.

In this context, the São Francisco River Integration Project (PISF) has emerged as the largest water infrastructure initiative in Brazil, aiming to benefit 12 million people across small, medium, and large cities in the semi-arid Northeast region. Two canals were constructed to achieve this goal: the North Axis, which supplies water to the hinterlands of Pernambuco, Ceará, Paraíba, and Rio Grande do Norte; and the East Axis, which directs water to parts of the Sertão and Agreste regions of Pernambuco and Paraíba^2^ ^,^ ^4^. 

However, the project has also raised public health concerns, particularly given its impact on the social and economic conditions of the population. These conditions may facilitate the spread of poverty-related diseases, such as schistosomiasis^2^ ^,^ ^3^ ^,^ ^5^.

According to the World Health Organization (WHO), *Schistosoma mansoni* schistosomiasis affects an estimated 240 million people across 78 countries, with approximately 700 million at risk of infection, mainly in Africa, Asia, and Latin America^5^ ^-^ ^7^.

Data from the National Survey on Prevalence of Schistosomiasis Mansoni and Geohelminthiasis Infections (INPEG), estimate that 3-4 million people in Brazil are infected with *S. mansoni*, with the Northeast region showing the highest prevalence (1.27%) compared to the national prevalence (0.99%), as well as the highest mortality rate (0.53 per 100,000 inhabitants, compared to the national rate of 0.22 per 100,000 in 2019)^8^ ^,^ ^9^. In addition to its morbidity burden, schistosomiasis is considered a neglected cause of death in Brazil^10^.

Environmental changes tend to shift the schistosomiasis transmission patterns, potentially contributing to the emergence of new endemic areas^11^. PISF is expected to cause substantial ecological modifications, which may enable the introduction and establishment of *Biomphalaria glabrata* (Say, 1818)-the most competent vector of schistosomiasis, in areas currently inhabited by other species such as *B. straminea* (Dunker, 1848). These alterations may lead to the displacement or decline of native snail populations, particularly under conditions influenced by environmental disturbances and human migration. Both *B. glabrata* and *B. straminea* have been reported in various regions of the São Francisco River basin^12^. The formation of new or altered aquatic habitats could promote ecological adaptation and spatial expansion of *B. glabrata*.

The issue of species introduction has been documented in other parts of the world^13^ ^-^ ^15^, including Brazil^16^ ^-^ ^18^.

Therefore, it is crucial to identify and monitor snail population dynamics in areas affected by PISF in Ceará. This strategic action seeks to provide evidence to support decision-making processes in local health policies aimed at preventing or mitigating potential impacts on the distribution of intermediate hosts, and consequently, the occurrence of schistosomiasis cases.

This study aimed to describe the distribution of freshwater malacofauna, including *S. mansoni* larvae across water bodies associated with the Meta 2N segment of the PISF in the state of Ceará.

## METHODS

This was a descriptive epidemiological study of freshwater malacological fauna in the state of Ceará conducted in March 2019. The study was conducted in the municipalities of Brejo Santo, Jati, and Mauriti located within the Meta 2N segment of the PISF. This section of the project extends over 39 kilometers and includes the construction of the Jati reservoir, expansion of the Atalho reservoir (both located in Jati), construction of the Porcos reservoir (between Jati and Brejo Santo), and construction of the Cana Brava, Cipó, Boi I, and Boi II reservoirs (all in Brejo Santo). This water infrastructure is interconnected by canals and aqueducts within the state of Ceará. Productive rural villages (PRV) were established to accommodate families displaced by land expropriation due to the PISF construction. The PRVs include Ipê (Jati: 14 houses), Descanso (Mauriti: 77 houses), and Vassouras (Brejo Santo: 154 houses). Each PRV is designed to support the productive activities of the resettled population, with each family receiving a dryland plot of approximately 6.0 hectares (ha) and an irrigated plot of approximately 1.0 ha. These PRVs are located within an area defined as environmentally impacted by the project according to the Environmental Impact Report^2^ ^,^ ^3^.

### Selection of sampling sites

The sampling locations for the survey of schistosomiasis intermediate hosts were selected based on their proximity to PRVs. Google Earth Pro^™^ was used to map each PRV, and a 2.5-km radius was drawn from the central point of each village to identify the nearby water bodies. A total of 35 water bodies were initially selected (13 from Jati, 11 from Brejo Santo, and 11 from Mauriti). All sites were visited with the support of local endemic disease control agents, and mollusks were collected only at sites with water and snails. Some selected sites were temporarily dry at the time of field visits due to the severe drought that occurred in Ceará at the time of the study. The geographic coordinates of each sampling site were recorded using a Garmin Montana^™^ 650 GPS device (UTM projection system) and stored in spreadsheets for subsequent thematic map creation.

### Snail collection

Mollusks were collected using a handheld dredge to scrape the submerged vegetation. The vegetation surface was also carefully examined for snails, particularly on the leaves and twigs, where small or juvenile specimens would be found. The collected mollusks were placed in labeled plastic containers, noting the location, type of breeding site, and collection date. The trap residues were thoroughly rinsed and inspected multiple times to ensure complete recovery of the mollusks before disposal. Each collection was conducted by two trained technicians within a demarcated 30 m² area over a 30-minute period.

For transportation, the snails were wrapped in moist gauze to maintain humidity, placed in plastic bags with proper labeling, and stored in rigid thermal containers. Subsequently, they were transported to the Parasitology and Mollusk Biology Research Laboratory at the Federal University of Ceará (LPMB-UFC), where they were kept in plastic containers with dechlorinated water and fed fresh, decontaminated lettuce^19^ until further analysis.

### *Schistosoma mansoni* cercariae shedding test

At the LPMB-UFC, *Biomphalaria* snails were individually exposed to artificial light (60 W lamp) at a distance of 30 cm for 4 hours to stimulate the shedding of *S. mansoni* cercariae or other medically relevant trematodes. The water from the containers was examined under a stereoscopic microscope. This process was repeated weekly for 30 days, following the technical procedures outlined in the Brazilian Ministry of Health's *Guide for the Surveillance and Control of Epidemiologically Important Mollusks* ^19^.

### Morphological classification

All the collected mollusks were analyzed and classified based on their external shell characteristics. Morphological techniques were used for species identification in collaboration with the National Reference Laboratory for Schistosomiasis - Malacology (LRNEM) at the Oswaldo Cruz Foundation (FIOCRUZ) Rio de Janeiro. Morphological identification at the genus level was performed using conchological features, whereas species-level identification was based on dissection and observation of the internal anatomy, following the protocol described by Silva et al. (2024)^20^.

### Classification of transmission risk of water bodies (WC)

Water bodies were evaluated according to the Brazilian Ministry of Health guidelines from the *Guide for the Surveillance and Control of Epidemiologically Important Mollusks* ^19^. The classification followed the summation of scores in fields 9 to 15 of Form PCE 103 from the Schistosomiasis Control Program, with the following categories: lower potential and contraindication of WC treatment, ≤14; Indication of WC treatment in the presence of snails and contamination, 15-17; and higher potential and indication of WC treatment, ≥18.

### Snail population density

The estimated snail population density was calculated according to the method described by Olivier and Schneiderman^21^, using the following expression:

$$Populationdensity=\frac{\frac{Numberofcollectedmollusks}{Numberofcollectors}}{Collectiontime}$$

### Environmental characterization

During the malacological survey, the environmental characteristics of the breeding sites were recorded and analyzed, including the type of water body, substrate, shading, associated fauna, and presence of other snail species. The pH, water temperature, and air temperature were measured using universal pH indicator paper (0-14, colorimetric scale) and a properly calibrated room thermometer.

### Statistical and geospatial analysis

Data on the sampled water bodies and collected snails were compiled using a spreadsheet. Spatial data were analyzed using the ArcGIS 9.3 software (Environmental Systems Research Institute, Redlands, CA, USA) for visualization and integration with descriptive attributes. Descriptive statistical analyses were performed using absolute and relative frequencies.

### Ethical approval

This study was approved by the Research Ethics Committee of the Federal University of Ceará (Nos. 2, 630, 278).

## RESULTS

### Malacological fauna

Of the 21 water bodies surveyed, 9 were located in Jati, 8 in Mauriti, and 4 in Brejo Santo. Freshwater mollusks of five genera, all from the class Gastropoda (Cuvier, 1795), were identified: *Biomphalaria* (Preston, 1910), *Melanoides* (Olivier, 1804), *Drepanotrema* (Crosse and Fischer, 1880), *Physa* (Draparnaud, 1801), and *Pomacea* (Perry, 1810). Additionally, Bivalvia spp. (Linnaeus, 1758) were observed in the water bodies of Jati and Brejo Santo.

Table 1 shows the distribution of freshwater snail species across the surveyed municipalities. *Biomphalaria* sp. and *Melanoides* sp. were the most prevalent and were recorded in 18 (85.7%) and 15 (71.4%) water bodies, respectively. In contrast, *Physa* spp. (2; 9.5%) and Bivalvia (4; 19.0%) exhibited a more limited distribution. The sites with the highest species richness and population density of *Biomphalaria* sp. were Lagoa da Fazenda do Mano (Mauriti) and Açude Atalho (Brejo Santo), followed by Lagoa da PRV Ipê (Jati). Conversely, some sites, including the reservoir used to supply water for construction and the Porcos dam in Brejo Santo, had no mollusks. The population density of planorbid snails was estimated according to the method proposed by Olivier and Schneiderman (1956)^21^.

**TABLE 1:** Freshwater snails distributed across collection sites in Jati, Mauriti, and Brejo Santo, State of Ceará, Brazil.

Municipality	Water Collection	Water Collection Name	Coordinates		***Biomphalaria* sp.**		Other Mollusks				
			Latitude	Longitude	Collected Quantity	Population Density	***Drepanotrema* sp.**	***Melanoides* sp.**	***Pomacea* sp.**	Classe Bivalvia	***Physa* sp.**
**Jati**	1	Jati Reservoir	7°42'17.72"	39° 0'20.78"	4	0.13		X	X		
	2	Jati Reservoir Bridge	7°42'8.39"	38°59'58.45"	8	0.27		X	X	X	
	3	Sabonete Stream / Porcos Stream	7°41'57.11"	38°59'46.41"	10	0.33		X			X
	4	Transposition Canal / Porcos Stream	7°41'44.64"	38°59'24.37"	39	1.3		X	X	X	
	5	Mr. Jacinto's Dam	7°41'39.83"	38°59'27.59"	18	0.6	X			X	X
	6	Barra Well	7°41'25.37"	38°59'47.07"	38	1.27		X	X		
	7	Vila Ipê Lagoon	7°41'48.27"	38°59'53.54"	70	2.33	X				
	8	Porcos Stream - Alto of Madalena	7°41'1.13"	39° 0'18.32"	23	0.77		X	X		
	9	Porcos Stream Bridge	7°41'8.51"	39° 0'31.49"	60	6		X	X		
**Brejo Santo**	1	Paulo Francisco Farm Dam	7°33'39.17"	38°54'29.16"	9	0.3				X	
	2	Dam near UBS Vieira	7°34'58.27"	38°53'34.19"	1	0.03	X				
	3	Porcos Dam	7°38'2.33"	38°53'18.24"	0	-		X			
	4	Atalho Dam	7°38'38.72"	38°53'42.70"	83	2.77		X	X		
**Mauriti**	1	Lagoon near Zé's house	7°27'18.31"	38°44'29.32"	22	0.73	X	X			
	2	Mano's Farm Dam	7°27'21.57"	38°44'47.03"	25	0.83		X			
	3	Works Supply Lagoon s	7°27'32.76"	38°44'51.07"	0	-		X			
	4	Mano's Farm Lagoon	7°27'25.94"	38°44'50.06"	266	26.6	X	X			
	5	Quixabinha Farm Dam	7°29'41.94"	38°45'7.16"	0	-		X			
	6	Wet Crossing on Gomes Dam Road	7°29'03.2"	38°44'30.5"	21	0.7					
	7	Gomes Dam	7°28'53.54"	38°43'42.40"	63	2.1	X		X		
	8	Fish Hatchery	7°28'54.73"	38°45'48.36"	5	0.17		X			

**FIGURE 1:**
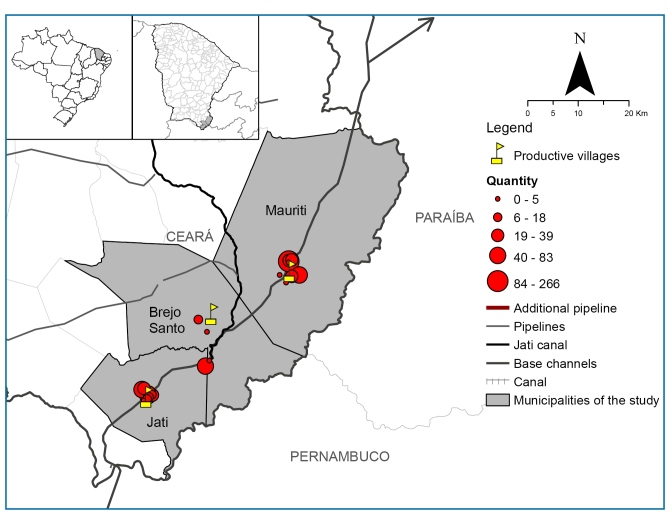
Distribution of *Biomphalaria* sp. according to a malacological survey in the municipalities of Jati, Mauriti, and Brejo Santo, State of Ceará, Brazil.

Figure 1 shows the geolocation distribution of sites where malacological surveys for *Biomphalaria* sp. were conducted, using proportional circles to represent abundance. All three municipalities had at least one site with *Biomphalaria* sp.. A total of 765 specimens were collected, with a mean of 43 individuals per site (range: 0-266). The site with the highest abundance was a pond on the “Mano” farm in Mauriti (266 individuals), followed by PRV Ipê in Jati (70 individuals).

### Classification of the transmission potential of water bodies

To classify the water bodies in terms of the schistosomiasis transmission potential, factors such as the frequency of population use, types of human activity, and presence of planorbid snails were considered. The common activities included washing clothes, dishes, and animals (80.9% of sites), and fishing (52.4%). Most water bodies were easily accessible (95.2%).

Based on these criteria, 38.1% of the water bodies (six in Jati and two in Mauriti) were classified as having a high epidemiological risk of transmission (Table 2) according to the guidelines of the Brazilian Ministry of Health.

**TABLE 2:** Potential transmission of water collections surveyed in the municipalities of Jati, Mauriti, and Brejo Santo, State of Ceará, Brazil.

Municipality	Water Collection	09 - Activity					10 - Access		11 - Water			12 - Snail		13 - Contamination		14 - User		15 - Assistance		Total
		1	1	4	4	1	1	4	1	3	3	1	3	1	2	1	2	1	2	
		Water Supply	Washing clothes, dishes, and animals	Fishing	Bodily hygiene	Swimming	No access	With access	Fast flow	Slow flow	Stagnant	Absent	Present	Absent	Present	Not infected	Infected	No	Yes	
**Jati**	1		X	X	X	X		X			X		X	X		X			X	18
	2		X	X	X	X		X			X		X	X		X			X	18
	3		X	X	X	X		X			X		X	X		X			X	18
	4		X	X	X	X		X		X			X	X		X			X	18
	5		X					X			X		X	X		X			X	15
	6		X					X			X		X	X		X			X	15
	7		X	X	X	X		X			X		X	X		X			X	18
	8		X	X	X	X		X			X		X		X	X			X	19
	9		X					X			X		X		X	X			X	16
**Brejo Santo**	1		X					X			X		X	X		X			X	15
	2		X					X			X		X	X		X			X	15
	3		X	X				X		X		X		X		X			X	16
	4			X	X	X		X	X				X	X		X			X	16
**Mauriti**	1		X					X			X		X	X		X			X	15
	2		X	X	X	X		X			X		X	X		X			X	18
	3							X			X	X		X		X			X	13
	4		X					X			X		X	X		X			X	15
	5		X	X	X	X		X			X	X		X		X			X	16
	6							X	X				X	X		X			X	12
	7		X	X	X	X		X			X		X	X		X			X	18
	8						X				X		X	X		X			X	11

### Species identification

Due to the limited number of collected specimens and high mortality during transport, species-level identification was performed only for samples from water bodies with high mollusk density.

Of the 97 *Biomphalaria* specimens collected and sent for analysis (60 from Mauriti, 27 from Brejo Santo, and 10 from Jati), 83 (87.4%) were examined at the National Reference Laboratory for Schistosomiasis - Malacology (LRNEM). Fourteen specimens died before the analysis.

Most specimens (96.4%) were identified as *Biomphalaria straminea* (Dunker, 1848). A few juveniles could not be definitively identified at the species level.

During dissection, some specimens were parasitized. The observed parasites included *Distomum brevifurcatum* pharyngeal forms characterized by simple-tailed cercariae, metacercariae, and sporocysts.

### Environmental conditions

Concurrently with the malacological survey, the characteristics of WC, such as the type of water body, substrate, shading, associated fauna, pH, water, and air temperature, were recorded and analyzed (Table 3).

**TABLE 3:** Description of the environment of the water collections studied in the municipalities of Jati, Mauriti, and Brejo Santo, State of Ceará, Brazil.

Municipality	Water Collection	Water pH	Water temperature (°C)	Ambient temperature (°C)	Type			Condition		Environment		Water			Substrate				Shading			Present fauna				
					Lakes and Dams	Streams, Rivers, and Creeks	Other	Perennial	Temporary	Natural	Artificial	Fast flow	Slow flow	Stagnant	Sand and stones	Mud	Muck	Plants	Completely	Partially	Absent	Fish	Mammals	Birds	Amphibians	Other
**Jati**	1	6	32.0	29.5			X	X			X			X	X	X	X	X			X	X		X		
	2	7	31.0	32.5			X	X			X			X			X	X		X		X				
	3	6	32.0	34.0			X	X			X			X			X	X		X		X	X			
	4	6	39.0	36.0			X		X		X		X			X	X				X	X	X			
	5	6	35.0	36.0	X				X		X			X			X	X		X		X			X	
	6	6	27.0	35.0		X		X		X				X		X	X	X		X		X				
	7	6	27.0	29.0	X				X	X				X			X	X	X							
	8	6	28.0	31.0		X		X		X			X		X					X		X				
	9	6	30.0	30.0		X		X		X				X			X			X		X				
**Brejo Santo**	1	6	30.0	29.5	X				X		X			X			X			X		X	X			
	2	7	36.5	34.0	X				X		X			X			X	X			X	X				
	3	6	33.5	35.0			X	X		X		X					X				X	X				
	4	6	33.0	36.0	X			X			X	X			X						X	X				
**Mauriti**	1	6	31.0	32.0	X				X		X			X		X	X	X			X	X				
	2	6	34.0	32.5	X			X			X			X			X				X	X				
	3	6	34.0	34.0	X				X		X			X			X			X		X				
	4	6	32.0	34.0	X				X		X			X			X				X					
	5	6	32.0	33.5					X		X			X			X	X			X	X		X		
	6	6	27.0	30.0			X		X		X	X			X					X		X				
	7	6	29.5	29.0	X				X		X			X	X			X		X						
	8	6	35.0	33.0			X	X			X			X			X				X	X				X

It is important to note that the most common environmental characteristics found were as follows: type of water resource, 41.6% lakes and reservoirs; condition, 52.4% temporary; environment, 76.2% artificial; and water, 76,2% stagnant flow. In addition, the average water temperature was 33.12 °C, and the ambient temperature was 32.64 °C.

## DISCUSSION

This study provides a current overview of the distribution of *Biomphalaria* snails, the intermediate hosts of *Schistosoma mansoni*, in the areas impacted by Brazil’s largest water infrastructure project, confirming the endemic nature of schistosomiasis in the surveyed municipalities and the potential for expanded transmission dynamics.

The potential direct impact of the PISF on *Biomphalaria* population density is associated with the construction of new reservoirs that promote organic matter accumulation due to the reduced water flow and facilitate the formation of new breeding sites^17^ ^,^ ^22^.

Snail biodiversity and distribution patterns in the studied municipalities were heterogeneous, even within microgeographic areas. Most surveyed water bodies were epidemiologically important breeding sites, providing ecological conditions favorable for *Biomphalaria* survival and used by the local communities for multiple purposes, including domestic use and income-generating activities^23^ ^-^ ^25^.

*Biomphalaria* snails are found in diverse aquatic habitats, including lentic and lotic environments, and prefer slow-flowing freshwater. They inhabit natural (e.g., ponds) and artificial (e.g., reservoirs) water bodies where the presence of macrophytes provides food, shelter, and egg-laying sites^26^ ^,^ ^27^.

The surveyed water bodies had pH values between 6.0 and 7.0. Acidic pH negatively affected calcium deposition in snail shells^28^. The semi-arid Northeast region of Brazil frequently experiences droughts, which drive snails to develop adaptive traits suited to flood-drought cycles^29^. *Biomphalaria* can enter anhydrobiosis (temporary suspension of metabolism) and diapause (dormant state), leading to morphological changes with species-specific adaptability; *B. straminea* is the most tolerant, whereas *B. tenagophila* is the least tolerant^30^.

Schistosomiasis and its most efficient vector, *B. glabrata*, persists in several municipalities along the São Francisco River Basin, whereas in Ceará, *B. straminea* is the predominant intermediate host^31^ ^,^ ^32^. Migration from endemic areas to construction zones, as well as the resettlement of families in 18 planned reservoir areas could reinforce the ecological and behavioral determinants of transmission, including the presence of infected individuals, competent snail hosts, suitable aquatic habitats, and water-contact practices^31^ ^-^ ^34^ ^,^ ^35^.

Consequently, currently disease-free areas where intermediate hosts are present may become endemic due to the environmental and socioeconomic changes driven by PISF. Unregulated occupation and degradation of natural ecosystems may create ideal conditions for snail proliferation and disease transmission^36^. Environmental disruptions caused by unsustainable development can accelerate disease emergence^37^ ^,^ ^38^.

Previous studies have documented *B. straminea* and other freshwater mollusks in Ceará^24^ ^,^ ^32^; however, monitoring efforts have historically been restricted to a few municipalities and limited in spatial scope. The present study expands the geographical coverage by incorporating additional localities affected by the PISF, including the areas surrounding the PRVs. This broader sampling approach provides new insights into the spatial distribution of the *Biomphalaria* species under the influence of large-scale hydrological modifications, and strengthens the evidence of potential ecological and epidemiological shifts in areas undergoing rapid environmental change.

Once operational, the PISF will supply water for various purposes, including human consumption, irrigation, and livestock, potentially increasing human contact with freshwater sources. These risks are amplified by the social vulnerability of local populations, marked by low education levels, subsistence economies, limited health coverage, and inadequate sanitation infrastructure^39^.

Although *B. straminea* shows low natural infection rates of *S. mansoni*, its high resistance to desiccation contributes to its wide distribution across the Northeast region of Brazil^40^.

Notably, *Biomphalaria* snails, which are vectors of schistosomiasis, also serve as the first intermediate hosts for other trematodes that may interfere with the biological cycle of *S. mansoni*, causing physiological changes in the transmitting snails and exhibiting antagonistic effects on the larval forms of this parasite^41^ ^,^ ^42^.

*Distoma brevifurcada faringeada (Brevifurcate pharyngeate distome)* are larvae found in gastropods and are produced in rediae by members of the *Clinostomidae* family, which are parasites of the oral cavity of birds. Metacercariae are produced in fish^43^ and can inadvertently infect humans by the consumption of raw fish containing metacercariae. *Clinostomum complanatum* has been reported to cause laryngopharyngitis, and ocular infection through a species of *Clinostomum* spp^44^ ^-^ ^46^.

This study has some limitations, primarily related to the severe drought in Ceará, which is restricted to only a few points in Brejo Santo. Two additional points outside the planned radius were included because of their epidemiological importance.

Despite these limitations, this study was conducted with methodological rigor, emphasizing the need to monitor the limnic malacofauna in the areas affected by PISF before full operationalization. It contributes to decision-making and reinforces the importance of continuous monitoring, not only for schistosomiasis, but also for other diseases linked to social vulnerability.

Other challenges for the effective control and monitoring of schistosomiasis and other neglected tropical diseases in these areas include discontinuity and limited access to diagnosis, treatment, and preventive services, as well as health promotion for these populations. Since the decentralization of the Schistosomiasis Control Program into primary healthcare in the late 1990s, earlier identification of risk areas and improved local monitoring have been enabled^28^. 

In Ceará, surveillance has expanded to previously unmonitored areas, focusing on locations with suspected cases and environmental conditions favorable for transmission. This facilitated the identification of new endemic foci^47^. Political and operational challenges persist, highlighting the need for collaboration across sectors to develop interventions that reduce schistosomiasis transmission.

The municipalities studied were focal points for schistosomiasis transmission in Ceará. Therefore, integrated health surveillance and care interventions involving systematic monitoring are critical. Interdisciplinary approaches under the One Health framework, which recognizes the connection between humans, animals, plants, and environmental health, are strategic for designing effective control measures in regions affected by major regional development water infrastructure projects.

This study highlights the heterogeneous distribution of *Biomphalaria* snails in municipalities affected by the São Francisco River Integration Project and emphasizes the creation of new breeding habitats due to environmental modifications. These findings underscore the importance of continued malacological monitoring in areas undergoing large-scale water infrastructure interventions to anticipate changes in the risk of schistosomiasis transmission and to inform targeted environmental and public health actions.

## Data Availability

The content is now available: http://vigilancia.saude.mg.gov.br/index.php/vigilancia-ambiental/
